# The changed occupation and behavioral among imported malaria cases 2009-2011 in Sukabumi District-West Java, Indonesia

**DOI:** 10.1186/1475-2875-11-S1-P128

**Published:** 2012-10-15

**Authors:** Dewi Susanna, Tris Eryando, Dian Pratiwi, Fajar Nugraha

**Affiliations:** 1 Department of Environmental Health, Faculty of Public Health, Universitas Indonesia, Kampus Ul Depok, Indonesia 16424; 2 Department Biostatistic and Health Informatics, Faculty of Public Health, Universitas Indonesia, Kampus Ul Depok, Indonesia 16424; 3 Center For Biostatistics and Health Informatics, Faculty of Public Health, Universitas Indonesia, Kampus Ul Depok, Indonesia 16424; 4 Center For Biostatistics and Health Informatics, Faculty of Public Health, Universitas Indonesia, Kampus Ul Depok, Indonesia 16424

## Background

A malaria outbreak occurred in the Sukabumi District in 2004, 785 cases were reported and 8 of them died. The imported cases are sometimes blamed as the trigger of the outbreak. Till now malaria has been endemic in Sukabumi. This research aimed to discover the characteristic of the migrants’ cases before and after they had got malaria.

## Materials and methods

The subjects were imported malaria cases that were collected from the Health Center in 4 Sub-district in Sukabumi District for the year 2009 to 2011, and 145 subjects were interviewed in their house using structured questionnaires. The data were analyzed descriptively to describe the distribution of the cases in terms of sex, occupation and the practice in preventing the transmission of malaria.

## Results

The majority of import malaria cases in Sukabumi were male and of productive age (more than 15 years old). They worked mostly in mining, and the rest were in plantation, merchant, and other type of labour or housewives. After they got infected by malaria they went or were sent back to their home land (Sukabumi). After they have got treatment and got well, some of them went back to their previous occupation in the same location and some in different location with the same activity. Few of them did not go back and stay unemployed in their home land.

In relation to the risk factors in malaria transmission, workers who were treated in Sukabumi mostly worked as miners and had experienced night shift, or some of them worked as “ojek” (motorcycle cab) and worked till late at night. Some of them had experienced night activities such as hanging out or watching television and some of them were not aware of malaria transmission. Around 56.6% used a mosquito net or repellent, and the rest did not take any protection to control malaria transmission.

## Conclusion

The imported malaria cases in Sukabumi were dominated by males of productive age, worked as miners experienced in night shift, without proper protection to avoid malaria transmission and they consistently looked to return to their previous occupation in the same location. So this is important for the district health office either in the home land or the work destination to promote malaria transmission protection among workers.

**Table 1:** The distribution of characteristic malaria import cases in Sukabumi, West Java, Indonesia 2009-2011

Total cases=145			
**Variable**	**Categories**	**Number**	**%**
Sex	Male	139	95.9%
	Female	6	4.1%
age	<15 years	0	0.0%
	15-54 years	138	95.2%
	> 54 years	7	4.8%
Occupation (before ill)	Plantation Growers	4	2.8%
	Merchants	4	2.8%
	Miners	130	89.7%
	Housewives	2	1.4%
	Casual Laborers	1	0.7%
	Other	4	2.8%
Occupation (after ill)	Moved out and change of occupation	35	24.1%
	Moved out and same occupation	10	6.9%
	Same location and change of occupation	12	8.3
	Same location and same occupation	85	58.6%
	Unemployed	3	2.1%
Night Behaviors	Toilet activity	1	0.7%
	Hangout, watch television	13	9.0%
	Patrolling	5	3.4%
	Fishing	4	2.8%
	Night shifts (miners, *ojek*)	82	56.6%
	No outside activity	4	2.8%
	Other	13	9.0%
The use of mosquito net and repellent	Use mosquito net and repellent	85	58.6%
	Do not use mosquito net and repellent	60	41.4%
